# Topics of Public Interest

**Published:** 1910-11

**Authors:** 


### TOPICS OF PUBLIC INTEREST

### Tuberculosis Crusader Praises Hughes.

**Homer Folks, Secretary of the State Charities Aid Association says Governor's Aid has advanced cause of White Plague Crusade.**

Homer Folks, Secretary of the State Charities Aid Association, the organisation which, in coöperation with the State Department of Health, is conducting the eminently successful crusade against tuberculosis in this state, gave out recently a statement praising Governor Hughes for his part in the crusade.

" The force of the Governor's personality and interest in this campaign," said Mr. Folks's statement, "has been felt all over the world." At the first meeting of the tuberculosis workers in this state in January, 1908, the Governor said:

If we had, through the misfortune of war, or the sudden rise of pestilence, or through some awful calamity, the destruction of life that annually takes place on account of the spread of tuberculosis, we should be appalled, and mass meetings would be held in every community and demand would be made that the most urgent measures should be adopted. It is only because we are accustomed to this waste of life and are prone to think that it is one of the dispensations of Providence that we go on about our business, little thinking of the preventive measures that are possible.

This statement has been a stimulus to the tuberculosis activity in practically every civilised country.

In each of the Governor's annual messages to the Legislature, there has been a recommendation for legislation to advance the cause of tuberculosis prevention. This has resulted in three years in state appropriation of $28,500 for the educational campaign that is being carried on by the State Department of Health in coöperation with the State Charities Aid Association.

The Governor has signed three of the most important health laws that have ever been put on the statute books in this state. The first one signed in 1908 makes it a misdemeanor for any physician not to report a case of tuberculosis and requires health officers to enforce proper supervision of the cases in the homes; the second one repealed the Bedell bill which put the final decision on the location of a hospital in the hands of town boards; the third and, perhaps, the most important, was passed last year and authorised boards of supervisors to make appropriations to build county tuberculosis hospital sanatoria. Governor Hughes also signed local legislation authorising expenditures for tuberculosis work in Syracuse, Buffalo, Niagara Falls, Lockport, Elmira, Yonkers, Newburg and Rensselaer and Dutchess counties, permitting an aggregate expenditure of more than $320,000.

The county hospital law has resulted in the establishment of or appropriations for ten county tuberculosis hospital sanatoria and the supervisors in fifteen other counties are investigating the subject.

Moreover, I regard the governor's interest in this campaign as having had a greater influence than that of any other person in arousing the enthusiasm which has resulted thus far in the establishment of nine camps, sixteen dispensaries, and the employment of thirty-five visiting nurses, and other relief measures which are daily helping to bring about " No Uncared-for Tuberculosis in New York State in 1915."

In referring to the fact that both the Republican and Democratic parties have incorporated in their platforms, planks pledging party support to the tuberculosis crusade, Mr. Folks said, " At the reception to the representatives of local committees affiliated with the State Charities Aid Association recently, Governor Hughes in one of the most earnest speeches that I have ever heard him deliver, said in effect, that no matter how men differ as to political theories they should work in harmony to drive tuberculosis out of existence. The two parties gave striking endorsement to this principle."

---

### Infantile Paralysis.

**Infantile Paralysis in New York State (Anterior Poliomyelitis)—Action of State Department of Health.**

Epidemic poliomyelitis has been added to the list of communicable diseases, the occurrence of which is required by the State Department of Health to be reported to local health officers and by them to the Department. Since 1881 medical literature has contained reports of outbreaks of infantile paralysis, and during the last five years these outbreaks in several parts of the world have increased in frequency out of proportion to the increased interest shown in the disease. That is, the increased number of reports cannot be attributed wholly to more accurate diagnosis or greater care in reporting the cases.

The disease is found to be more prevalent in cold than in warm countries. More cases have been reported from the northern part of the United States than from any other part of the world. It occurs mostly in children, but many adults have been afflicted. In 1907 there was an epidemic of 2,500 cases in New York, the largest ever reported. It generally begins late in the summer and ends after a few hard frosts, in October.

Laboratory workers have recently demonstrated that infantile paralysis is an infectious disease caused by a living organism so small that it can pass through a bacterial filter. It is thought to be most contagious during the early, or febrile stage of the disease. Most of the laboratory study has been made upon monkeys who acquire the disease by inoculation of an emulsion of certain tissues from a human being dying of the disease, and from affected monkeys. The laboratory has not yet put us in possession of the essential facts as to how the disease enters the human body, nor how to prevent or check its spread. The laboratory work must be supplemented by a field study. We need to classify the early symptoms and establish, if there be such, types of the disease; we need to investigate the conditions under which infection seems to occur, with a special inquiry as to the mode of entry of the virus into the body.

The New York State Department of Health desires the co-operation of the physicians of the State in the making of such an investigation. Valuable work upon the same lines has already been done by the medical profession and the State Board of Health of Massachusetts, and for the sake of uniformity and with a view to ease of comparative study, the New York State Department of Health is sending out to the Health Officers for distribution among the physicians of the State, a duplicate of the blank used by the Massachusetts Board in its investigation. It is sincerely hoped that every physician in whose practice a case of infantile paralysis may occur, will use this form and make as full and prompt a report as possible to the State Department of Health.

With a view to the prevention of the disease, the State Department of Health expects that every case discovered will be quarantined. Some local boards of health have already passed an ordinance requiring a quarantine in this disease, and such an action is approved by the department. The discharges from a patient—stools, urine, sputum—should be disinfected.

---

### Plan to Spend a Million.

**Tuberculosis Association Shows how Money from Red Cross Christmas Seals Would Provide Needed Hospitals.**

What "A Million for Tuberculosis from Red Cross Seals" will do in the checking of consumption, is explained in a bulletin issued today by the National Association for the Study and Prevention of Tuberculosis.

Counting every available bed for consumptives in the United States, even those in almshouses, penal institutions, and hospitals for the insane, there are at the present time accommodations for hardly 30,000 tuberculosis patients. This is just about one bed for every ten indigent consumptives, and if all tuberculous persons in the country are counted, both rich and poor, hardly one for every twenty-five or thirty. If sufficient hospital accommodations are provided only for those who are too poor to pay the full price for their treatment, fully 275,000 more beds in special institutions for tuberculosis would be needed at once. The immense outlay necessary to provide and maintain so many beds in hospitals, makes it imperative, the National Association for the Study and Prevention of Tuberculosis declares, that such institutions be erected from public money, either municipal, county or state. In order to get appropriations for public hospitals for tuberculosis, agitation is necessary, and in order to create a campaign of agitation, organisation is demanded. But in order that an organisation may carry on an effective campaign, funds are needed.

These funds it is proposed to secure in as many communities as possible from the sale of Red Cross Seals. The National Association cites one illustration of the way in which a small sum spent in education has secured large appropriations. The New York State Charities Aid Association in the three years, 1908, 1909, and 1910 has spent in the up-state portion of New York about $55,000 in arousing the people to the dangers of tuberculosis. As a direct result of the public sentiment produced by this outlay, the state, county, and municipal authorities have already appropriated for tuberculosis work $1,500,000 and appropriations for hundreds of thousands of dollars are pending. Hundreds of hospital beds have been provided, and the Association already aims for " No Uncared for Tuberculosis in 1915."

Thus, the National Association says if a million dollars is realised from the sale of Red Cross Seals, millions more will be added to it from the public treasuries. Last year 25,000,000 stamps were sold. It is aimed this year to sell four times as many.

---

### Public Health News.

Furnished by W. W. MILLS, West New Brighton, N. Y.

That there are 4,200,000 persons sick in this country at the present time; that 1,500,000 will die within the year; that 5,000,000 homes, or 25,000,000 people, a quarter of the country's population, are more or less wretched because of sickness or death; that one out of every twelve people living today will die from tuberculosis—these are some of the statements made by Prof. J. Pease Norton, of Yale, in the first issue of the " Popular Insurance Magazine."

He cites these cold and bare facts, not to alarm, but to stir the public to action that will compel the federal government to protect human life against disease. He points out that the Department of Agriculture spends $7,000,000 a year on plant health and animal health; that it saves hogs from cholera, and elm trees from beetles, but does not protect human lives from disease.

### LIFE WASTE UNCHECKED.

Every domestic animal of the farm has a better chance to live because of the great work done by the Department of Agriculture in the past ten years at a cost of $50,000,000, but, Dr. Norton points out, 6,000,000 of the people now living in this country will be carried off by diseases of the heart or kidneys, 8,000,000 will perish from pneumonia, and within a decade 6,000,000 babies will die before they reach the age of two years. Dr. Norton adds:

And yet this number could probably be decreased by as much as one-half. But nothing is done.

Of all the grievous wastes of nations, death, war, disease, accidents, vice, crime, fire, and bad government, by all odds the heaviest burden to the individual and to society, as a whole, is caused by the life wastes.

The life wastes are the measures of the suffering caused by preventable death, preventable disease and preventable accidents. In comparison with the life wastes all other wastes of nations pale into insignificance.

### HIGH COST OF INSURANCE.

It is because of this unchecked life waste, Dr. Norton shows, that the cost of life insurance is so high. He says:

If the cost of insurance could be reduced to one-half the present rates by preventing the spread of disease, by preventing needless death, and by preventing needless accidents to a greater extent than at present—and most competent experts have testified that this is possible—the policyholders could buy with the same premiums twice as much insurance.

Such a reduction in rates would be followed by an extension of insurance in all directions on a scale almost beyond our present comprehension. For insurance and public health organisation are society's chief weapons against the life wastes.

### LEAGUE OF POLICY HOLDERS.

To effect, by concerted action, the saving of lives and the consequent reduction in the cost of insurance, Dr. Norton has formed a League of the Policy Holders of America to wage warfare against the preventable wastes of death, disease, accidents, fire and crime. Its headquarters are at 34 West Thirty-third street, New York. Dr. Norton says:

Because the government is not efficient in prevention of these wastes, the policyholders of America are obliged to pay year after year higher premiums than they would under an efficient health organisation.

More than a majority of all voters are policyholders. All that the policyholders need to do is to make their wishes known to their political representatives.

With the watchword, "Prevention, not remedy," Dr. Norton plans to organise the policy holders in a great campaign that will not only help themselves financially by reducing the cost of insurance, but will at the same time do a mighty good to the nation in gaining for all longer and happier lives.

New York, October 13, 1910.

---

### Civil Service Examination

PHYSICIAN (MALE).—November 23, 1910.—The United States Civil Service Commission announces an examination on November 23, 1910, at the usual places, to secure eligibles from which to make certification to fill vacancies as they may occur in the position of physician in the different services. As an insufficient number of eligibles to meet the needs of the Indian and Isthmian Canal services resulted from the examination held on September 14, qualified persons are urged to enter this examination. Full information regarding this examination is contained in section 197 of the Manual of Examinations, revised to July 1, 1910.

Applicants should at once apply for a copy of the Manual and the proper forms either to the United States Civil Service Commission, Washington, D. C., or to the secretary of the board of examiners as follows: Post-office, Boston, Mass., Philadelphia Pa., Atlanta, Ga., Cincinnati, Ohio, Chicago, Ill., St. Paul, Minn., Seattle, Wash., San Francisco, Cal.; custom house, New York, N. Y., New Orleans, La.; old custom house, St. Louis, Mo. No application will be accepted unless properly executed and filed with the Commission at Washington. In applying for this examination the exact title, as given at the head of this announcement, should be used in the application.

As examination papers are shipped direct from the Commission to the places of examination, it is necessary that applications be received in ample time to arrange for the examination desired at the place indicated by the applicant. The Commission will therefore arrange to examine any applicant whose application is received in time to permit the shipment of the necessary papers.

